# Development of a novel tool: a nomogram for predicting in-hospital mortality of patients in intensive care unit after percutaneous coronary intervention

**DOI:** 10.1186/s12871-022-01923-y

**Published:** 2023-01-06

**Authors:** Miao Yuan, Bin Cheng Ren, Yu Wang, Fuxian Ren, Dengfeng Gao

**Affiliations:** 1 Cardiology diseases department, Xi’an Jiaotong University Second Affiliated Hospital, NO.157 Xiwu Rd, Xi’an, China; 2 Department of Cardiology, Meishan Brach of the Third Affiliated Hospital, Yanan University School of Medical, Meishan, Sichuan People’s Republic of China

**Keywords:** PCI, In-hospital mortality, Nomogram

## Abstract

**Backgrounds:**

Increased risk of in-hospital mortality is critical to guide medical decisions and it played a central role in intensive care unit (ICU) with high risk of in-hospital mortality after primary percutaneous coronary intervention (PCI). At present，most predicting tools for in-hospital mortality after PCI were based on the results of coronary angiography, echocardiography, and laboratory results which are difficult to obtain at admission. The difficulty of using these tools limit their clinical application. This study aimed to develop a clinical prognostic nomogram to predict the in-hospital mortality of patients in ICU after PCI.

**Methods:**

We extracted data from a public database named the Medical Information Mart for Intensive Care (MIMIC III). Adult patients with coronary artery stent insertion were included. They were divided into two groups according to the primary outcome (death in hospital or survive). All patients were randomly divided into training set and validation set randomly at a ratio of 6:4. Least absolute shrinkage and selection operator (LASSO) regression was performed in the training set to select optimal variables to predict the in-hospital mortality of patients in ICU after PCI. The multivariate logistical analysis was performed to develop a nomogram. Finally, the predictive efficiency of the nomogram was assessed by area under the receiver operating characteristic curve (AUROC)，integrated discrimination improvement (IDI), and net reclassification improvement (NRI), and clinical net benefit was assessed by Decision curve analysis (DCA).

**Results:**

A total of 2160 patients were recruited in this study. By using LASSO, 17 variables were finally included. We used multivariate logistic regression to construct a prediction model which was presented in the form of a nomogram. The calibration plot of the nomogram revealed good fit in the training set and validation set. Compared with the sequential organ failure assessment (SOFA) and scale for the assessment of positive symptoms II (SAPS II) scores, the nomogram exhibited better AUROC of 0.907 (95% confidence interval [CI] was 0.880-0.933, *p* <  0.001) and 0.901 (95% CI was 0.865-0.936, *P* <  0.001) in the training set and validation set, respectively. In addition, DCA of the nomogram showed that it could achieve good net benefit in the clinic.

**Conclusions:**

A new nomogram was constructed, and it presented excellent performance in predicting in-hospital mortality of patients in ICU after PCI.

**Supplementary Information:**

The online version contains supplementary material available at 10.1186/s12871-022-01923-y.

## Background

Coronary heart disease (CHD) has a very high morbidity and mortality rate worldwide [1–3]. Primary percutaneous coronary intervention (PCI) is one of the most important treatment strategies for coronary artery disease [4–7]. .However, many patients still have poor outcomes after PCI, including acute heart failure, stroke, and even death [8]. Early detection of high-risk patients is crucial and rapid intervention could impact the outcome. Therefore, we need to identify some indicators or models to assess the risk of death of patients after PCI. For clinicians, this model should be simple and accurate, as it is more suitable for clinical practice.

At present, there are many traditional tools, such as the Framingham risk score and Thrombolysis in Myocardial Infarction (TIMI) risk score, used to predict the prognosis of CHD patients [9, 10]. The significance of these evaluation tools is to enable clinicians to identify high-risk patients as soon as possible. Focusing on high-risk patients can make the work of clinicians more targeted. In acute myocardial infarction (AMI) patients, many new models could be used to predict the risk of death based on clinical indicators and coronary angiography [11–13]. With the development of machine learning, several studies have constructed nomograms for ST-segment elevation myocardial infarction patients to predict the risk of death [14, 15]. However, most of these studies focus on patients with acute coronary syndrome (ACS) and were based on the results of coronary angiography, echocardiography, and laboratory results, which are difficult to obtain at admission. The difficulty of using these models limits their clinical application. A study indicates that the SOFA and SAPS II can predictors of survival following acute myocardial infarction-related refractory cardiogenic shock, but did not perform well [16]. Although the SOFA and SAPS II scoring system are widely used in intensive care unit (ICU), both of them reflect the patient’s general condition and not specific to patients after PCI and has limitations in evaluating the risk of in-hospital death of patients after PCI.

Simple clinical indicators such as vital signs, white blood cell (WBC), hemoglobin, platelets, potassium in serum, sodium in serum, prothrombin time (PT) and MB isoenzyme of creatine kinase (CKMB) are easy to obtain. They did not need complex and expensive equipment and these results are objective. We were committed to using simple clinical indicators to develop a model for predicting the risk of in-hospital death in patients in ICU after PCI. To the best of our knowledge, no previous study had investigated the ability to develop simple nomogram for prediction of in hospital mortality of patients in ICU after PCI.

## Material and methods

### Data source

All the patient data used in our study came from an online international database—Medical Information Mart for Intensive Care III (MIMIC III) (version 1.4). MIMIC III is a large, single-center database comprising information related to patients admitted to critical care units of Beth Israel Deaconess Medical Center. It included patients who were admitted to the ICU between 2001 and 2012. This database includes a total of 53,423 different admission data points. MIMIC III contains a wealth of clinical information, including vital signs, demographic information, laboratory results, treatment records and nursing records. The Institutional Review Boards at both Massachusetts Institute of Technology and the Beth Israel Deaconess Medical Center approved the use of the data for research. The database can be accessed by certified researchers, so no additional informed consent of the patient and ethical approval are required [17]. The certified researcher of this study is Miao Yuan (no. 7382002).

### Study population and data extraction

Adult patients with the diagnosis of insertion of coronary artery stents based on the International Classification of Diseases (ICD)-9 code were selected (ICD-9 codes were 3606 and 3607). We excluded patients who were younger than 18 years old. If a patient had information from more than one admission, we only considered the first ICU information.

We used Structured Query Language and PostgreSQL software (version 10.0) to obtain data from MIMIC III. These data included demographic characteristics (including age and sex), SOFA score, Elixhauser Comorbidity Index, SAPS II score, body mass index (BMI), mean value of vital signs during the first 24 hours of ICU stay (including heart rate, systolic pressure, diastolic pressure, and respiration rate), treatment (ventilation treatment type and the use of vasoactive drugs), laboratory results (including hemoglobin, platelets, sodium, potassium, PT, WBC, CKMB, anion gap, bicarbonate, chloride, troponin T (TnT), troponin I (TnI), low-density lipoprotein (LDL), triglyceride (TG), and lactate (Lac)), the type of coronary artery stent (drug eluting stent or non-drug eluting stent), with or without the diagnosis of AMI. We extracted vital signs and laboratory results within 24 hours after admission, and if patients had multiple results in the first 24 hours, we took their average.

Elixhauser Comorbidity Index is a method of categorizing comorbidities of patients based on the ICD diagnosis codes found in administrative data, such as hospital abstracts data. Each comorbidity category is dichotomous (the comorbidity either present or it is not). The Index can be used to predict hospital resource use and in-hospital mortality [18].

In our study, the endpoint was died in hospital. All variables with more than 20% missing values were deleted (including BMI, TnT, TnI, LDL, TG, and Lac). If the missing value of a continuous variable was less than 10%, the missing value was filled by multiple interpolation.

## Statistical analysis

We used the Kolmogorov-Smirnov test to test the distribution of variables. Continuous normally distributed variables were represented by the mean ± standard deviation, while continuous nonnormal distribution were represented by the median (interquartile range). Categorical variables were expressed as numbers (percentages). If the variable conformed to normal distributions, the baseline characteristics among the two groups (survive group and died in hospital group) were compared using the unpaired student’s t test, and continuous variables with nonnormal distributions were compared by the Mann-Whitney U test. The differences of the ratio of survive between groups of categorical variables were evaluated by the chi-square test. When comparing the categorical variables between groups, if the theoretical frequency was less than 5, Fisher’s exact test was used for analysis.

We used without replacement sampling (the sample function in R) to randomly select 60% of all patients as training set and the last 40% as validation set. The data of the training set were used to build the prediction model, and the validation set was used to verify the model.

We used the least absolute shrinkage and selection operator (LASSO) to select the optimal predictive risk factors for in-hospital mortality after PCI. LASSO implements the selection of variables by applying the regularization process. A penalty term is added to the coefficient of the model on the ordinary linear square, and a more refined model is obtained by constructing the penalty function [19].We assigned all covariable to different penalties. The penalty term λ of each covariable is optimized by 10 times cross-validation. The regression coefficient of the covariable with less correlation shrinks to zero by adding the penalty term λ. The covariates in which the penalty term λ is not zero are further incorporated into the construction of the prediction model.

The factors selected by LASSO regression were incorporated into the prediction model. The 95% confidence interval of the odds ratio (OR) value and the *p* value were used to evaluate the variables. The level of statistical significance was two-sided. We used multivariable logistic regression to construct a prediction model. The model was provided in the form of a nomogram.

The nomogram was compared with the SOFA and SAPS II. The sensitivity and specificity of the three models were used to generate a receiver operating characteristic (ROC) curve of each model, and the area under the receiver operating characteristic curve (AUROC) was calculated. The AUROC values and 95% confidence intervals were used to compare the different prediction models to confirm which model had better prediction ability. The comparison method was “Delong”. In addition to the AUROC, we used the net reclassification improvement (NRI) and integrated discrimination improvement (IDI) to compare the predictive value between the nomogram and the SOFA or SAPS II. The calibration of the nomogram was evaluated by plotting the calibration curve. Decision curve analysis (DCA) could be performed by quantifying the net benefit under different threshold probabilities. DCA was used to evaluate the net benefit of nomogram in predicting the in-hospital death of patients after PCI. In the DCA diagram, the probability of patient is in hospital death is Pi; when Pi reaches a certain threshold (Pt), it is positive and treatment taken. There will be benefits from treatment of patients, as well as harm from treatment of non-patients and loss (disadvantages) from patient non-treatment [20].

.Statistical analysis was performed using R version 4.0.3. We used the ‘glmnet’ R package to implement the LASSO regression model and the ‘rms’ package for the mortality risk prediction nomogram.

## Results

### Characteristics of the patients included in the study

A total of 2160 patients were enrolled in the study according to the inclusion and exclusion criteria. The screening process is shown in Additional file 1. All patients were randomly divided into training set and validation set according to the ratio of 6:4. The training set contained 1296 patients, and the validation set contained 864 patients. All continuous variables didn’t conform to a normal distribution(*p* <  0.01) and were represented by the median (interquartile range). The demographic characteristics of all patients at baseline are shown in Table 1. The demographic characteristics of the training set and the validation set are shown in Additional file 2 and Additional file 3, respectively.

**Table 1:** Baseline characteristics of patients

Variable	Survive ***n*** = 1968	Died in hospital ***n*** = 192	Rate (died in hospital /total)	***p***-value
Age(years)	69 (58-79)	76 (66-83)	–	*p* < 0.001
Gender(male/female)	1272/696	102/90	–	0.002
Risk score				
SOFA	1 (0-3)	6 (3-9)	–	*p* < 0.001
SAPS II	29 (23-37)	48 (37-58)	–	*p* < 0.001
Elixhauser comorbidity index	0 (0-5)	5 (0-10)	–	*p* < 0.001
Vital parameters				
Systolic blood pressure (mmHg))	113 (105-124)	103.5 (93-115)	–	*p* < 0.001
Diastolic blood pressure (mmHg)	60 (53-67)	54 (49-60)	–	*p* < 0.001
Heart rate(min^−1^)	76 (68-86)	86 (75-100)	–	*p* < 0.001
Respiratory rate(min^−1^)	18 (16-20)	20 (17-22)	–	*p* < 0.001
Laboratory results				
Hemoglobin (g/dL)	12 (10-13)	11 (10-12)	–	*p* < 0.001
Platelet (×10^9^/L)	220 (180-270)	212.5 (158.25-270)	–	0.024
Potassium (mmol/L)	4.10 (3.8-4.4)	4.3 (3.9-4.7)	–	*p* < 0.001
Sodium (mmol/L)	139 (137-140)	137 (134-140)	–	0.001
PT (s)	13 (13-15)	15 (14-17)	–	*p* < 0.001
WBC (×10^9^/L)	11 (9-14)	13 (10-17.75)	–	*p* < 0.001
CKMB (mmol/L)	69 (17-203)	82.5 (18-206)	–	0.341
Anion gap (mmol/L)	14 (12-15.5)	17 (14.5-19.38)	–	*p* < 0.001
Bicarbonate (mmol/L)	24 (22-26)	21 (17-23)	–	*p* < 0.001
Chloride (mmol/L)	104 (102-107)	104 (100-108)	–	0.934
the type of coronary stent, *n* (%)				0.001
Non-drug-eluting stent	964 (48.98%)	137 (71.35%)	12.44%	
Drug-eluting stent	1004 (51.02%)	55 (28.65%)	5.19%	
AMI, *n* (%)				0.65
Without the diagnose of AMI	940 (47.76%)	95 (49.48%)	9.18%	
With the diagnose of AMI	1028 (52.24%)	97 (50.52%)	8.62%	
Ventilation treatment type, *n* (%)				*p* < 0.001
None	196 (9.96%)	3 (1.56%)	1.51%	
oxygen therapy	1733 (88.06%)	118 (61.46%)	6.37%	
NIMV	28 (1.42%)	69 (35.94%)	71.13%	
IMV	11 (0.56%)	2 (1.04%)	15.38%	
Vasoactive drug, *n* (%)				*p* < 0.001
None	1276 (64.84%)	36 (18.75%)	2.74%	
Vasopressin	1 (0.05%)	0	0	
Dobutamine	12 (0.61%)	0	0	
Epinephrine	45 (2.29%)	0	0	
Phenylephrine	106 (5.39%)	10 (5.21%)	8.62%	
Dopamine	190 (9.65%)	27 (14.06%)	12.44%	
Norepinephrine	61 (3.10%)	16 (8.33%)	20.78%	
Any two vasoactive drugs	188 (9.55%)	51 (26.56%)	21.34%	
Any three vasoactive drugs	32 (1.63%)	20 (10.42%)	38.46%	
Any four or more than four vasoactive drugs	57 (2.90%)	32 (16.67%)	35.96%	

*SOFA* sequential organ failure assessment, *SAPS II* scale for assessment of positive symptoms II, *PT* prothrombin time, *WBC* white blood cell count, *CKMB* MB isoenzyme of creatine kinase, *AMI* acute myocadiac infraction, *NIMV* noninvasive mechanical ventilation, *IMV* invasive mechanical ventilation

### Univariate analysis

After PCI, the patients who died in the hospital were older than those who survived, with a median age of 76 years (66-83 years) for patients who died in the hospital and 69 years (58-79 years) for surviving patients. There were more males (63.6%) than females undergoing PCI(*p* <  0.001). However, among female patients, the risk of in-hospital mortality was 11.45% higher than that of male patients (7.42%). The SOFA score, SAPS II score, and Elixhauser Comorbidity Index were higher in patients who died in the hospital than in survivors (*p* <  0.001). In addition, patients who died in the hospital had lower systolic blood pressure and diastolic blood pressure and a higher heart rate and respiratory rate (*p* <  0.05). Patients who died in the hospital were more likely to have lower hemoglobin, platelets, sodium, and bicarbonate levels and higher potassium, PT, WBC, and anion gaps (*p* <  0.05). The levels of CKMB and chloride were not significantly different between the patients who died in the hospital and those who survived (*p* = 0.341, *p* = 0.934). The death rate was 12.44% in patients with non-drug-eluting stents and was higher than the 5.19% rate in patients with drug-eluting stents(*p* <  0.001). There is no significant difference of the in-hospital mortality between the AMI patients (8.62%) and non-AMI patients (9.18%) (*p* = 0.650).

Under different types of mechanical ventilation treatment, patients using noninvasive mechanical ventilation (NIMV) had the highest mortality rate (71.13%), followed by those using invasive mechanical ventilation (IMV) (15.39%), oxygen therapy (6.38%) and none (1.51%). The difference between the four treatments was significant (*p* <  0.001). Patients who used multiple vasoactive drugs at the same time had a higher risk of death in the hospital (*p* <  0.001).

### Selection of the risk predictors

**Fig. 1:**
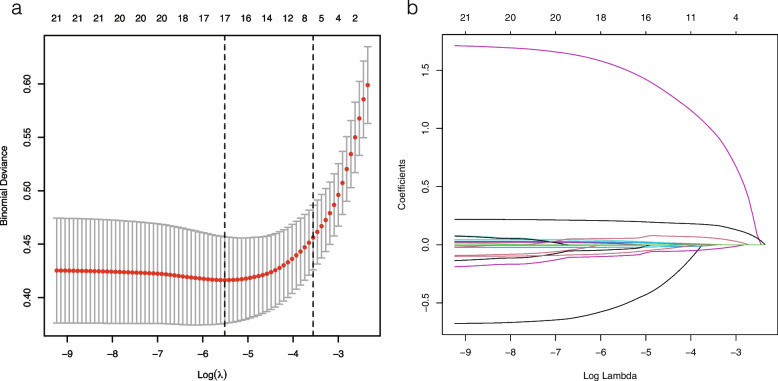
Use of the LASSO regression to select the variables in the training set. **a** We use the method of 10 times cross-validation to select the optimal value of λ. The value of parameter λ is determined by the mean square error. We chose the λ value with the lowest mean square error. The λ value was 0.004. **b** Twenty-two variables were screened by LASSO regression, among which the optimal λ resulted in 17 nonzero coefficients

17 risk predictors were screened by LASSO regression, including the type of coronary stent, age, Elixhauser Comorbidity Index, ventilation treatment type, use of vasoactive drugs, heart rate, systolic pressure, diastolic pressure, respiratory rate, bicarbonate, chloride, hemoglobin, platelets, PT, WBC, CKMB, and anion gap were selected as risk predictors of in-hospital mortality in patients after PCI (Fig. 1a, b).

### Development of a prediction nomogram

**Fig. 2:**
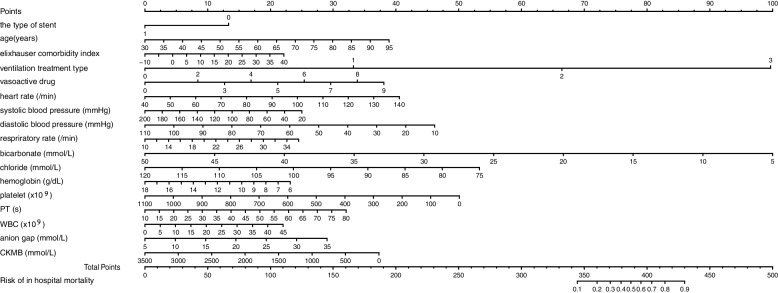
Nomogram for predicting in-hospital mortality in patients with PCI. Each variable has a coordinate axis, and by drawing a vertical line through the corresponding point on this coordinate axis, the point of intersection with the topmost scoring axis is the score of the variable. Adding all the variable scores together gives the in-hospital mortality probability corresponding to the total score at the bottom of the nomogram. In the type of stent option, “0” represents a non-drug-eluting stent and “1” represents a drug-eluting stent. In the ventilation treatment type options, “0” represents none, “1” represents oxygen therapy, “2” represents noninvasive mechanical ventilation, and “3” represents invasive mechanical ventilation. In the vasoactive drug option, “0” represents none, “1” represents vasopressin, “2” represents dobutamine, “3” represents epinephrine, “4” represents phenylephrine, “5” represents dopamine, “6” represents norepinephrine, “7” represents the use of any two vasoactive drugs, “8” represents the use of any three vasoactive drugs, and “9” represents the use of any four or more vasoactive drugs

The results of the multivariable logistic regression analysis among the risk predictors are given in Table 2. Based on the multivariate logistic regression analysis, the predicting equation was generated with the calculated coefficients: logit(1/1 − *P*) = 1.617 − 0.685*the type of stent+ 0.031*age+ 0.023*Elixhauser comorbidity index+ 1.706*ventilation+ 0.217*vasoactive drug+ 0.021*heart rate− 0.007*systolic blood pressure− 0.024*diastolic blood pressure+ 0.048*respiratory rate− 0.114*bicarbonate− 0.061*chloride− 0.099*hemoglobin− 0.002*platelet+ 0.024*PT + 0.025*WBC + 0.050*anion gap-0.001*CKMB. A nomogram representing the predicting model was established according to the variables and their corresponding regression coefficients (Fig. 2).

**Table 2:** Prediction factors for in-hospital mortality in patients after PCI

Intercept and variable	***p*** value	regression coefficient	Odds ratio (95% CI)
the type of stent	0.012	-0.685	0.504 (0.291-0.851)
age	0.005	0.031	1.031 (1.010-1.054)
Elixhauser comorbidity index	0.307	0.023	1.023 (0.979-1.068)
ventilation treatment type	< 0.001	1.706	5.507 (3.029-10.199)
vasoactive drug	< 0.001	0.217	1.243 (1.151-1.345)
heart rate	0.025	0.021	1.021 (1.003-1.040)
systolic blood pressure	0.438	-0.007	0.993 (0.975-1.011)
diastolic blood pressure	0.133	-0.024	0.977 (0.947-1.007)
respiratory rate	0.161	0.048	1.050 (0.980-1.123)
bicarbonate	0.005	-0.114	0.892 (0.822-0.965)
chloride	0.020	-0.061	0.941 (0.893-0.991)
hemoglobin	0.201	-0.099	0.906 (0.777-1.054)
platelet	0.119	-0.002	0.998 (0.995-1.001)
PT	0.255	0.024	1.024 (0.979-1.063)
WBC	0.363	0.025	1.025 (0.971-1.081)
anion gap	0.250	0.050	1.051 (0.965-1.145)
CKMB	0.310	-0.001	0.999 (0.998-1.001)
intercept	0.697	1.617	

*PCI* percutaneous coronary intervention, *PT* prothrombin time, *WBC* white blood cell count, *CKMB* MB isoenzyme of creatine kinase

### Verification of the predictive value of the nomogram

**Fig. 3:**
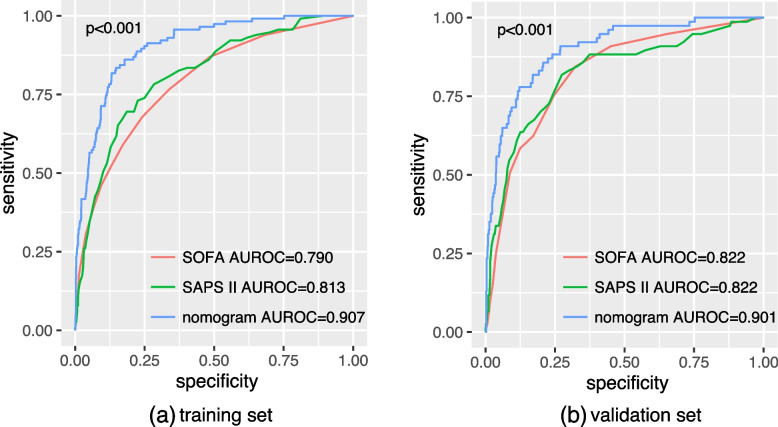
The ROC curve of the nomogram, SOFA, and SAPS II. **a** Training set, **b** validation set

AUROC, IDI, and NRI to compare the nomogram with the SOFA and SASP II for predicting the in-hospital risk of mortality in critical ill patients after PCI are shown in Table 3. Comparing the nomogram with SOFA in the training set and validation set, both IDI and NRI higher than 0 indicated that the prediction efficiency of the nomogram was higher than that of SOFA (*p* <  0.001). The same results were obtained by comparing the nomogram with SAPS II (*p* <  0.001). In Fig. 3, the results showed that regardless of the training set or validation set, the AUROC of the nomogram was significantly higher than that of SOFA and SAPS II (*p* <  0.001). This means that the nomogram performed better than SOFA and SAPS II in predicting the risk of in-hospital mortality of critical ill patients after PCI.

**Table 3:** Comparison of different models in predicting the in-hospital mortality of patients after PCI

	Predictive Model	AUROC	***p*** value	IDI	***p*** value	NRI	***p*** value
Training set	nomogram	0.907 [0.880-0.933]					
	SOFA	0.790 [0.746-0.835]	< 0.001	0.181 [0.139-0.224]	< 0.001	0.964 (0.645-1.227)	< 0.001
	SASP II	0.813 [0.772-0.855]	< 0.001	0.176 [0.126-0.226]	< 0.001	0.663 (0.435-1.029	< 0.001
Validation set	nomogram	0.901 [0.865-0.936]					
	SOFA	0.822 [0.774-0.870]	< 0.001	0.191 [0.131-0.251]	< 0.001	0.848 (0.405-1.194)	< 0.001
	SASP II	0.822 [0.768-0.876]	< 0.001	0.152 [0.087-0.217]	< 0.001	0.601 (0.196-1.032)	< 0.001

The *p* value of IDI and NRI were calculated by comparing the nomogram with SOFA or SAPS II

*PCI* percutaneous coronary intervention, *SOFA* Sequential Organ Failure Assessment, *AUROC* area under the receiver operating characteristic curve, *IDI* integrated discrimination improvement, *NRI* net reclassification improvement

**Fig. 4:**
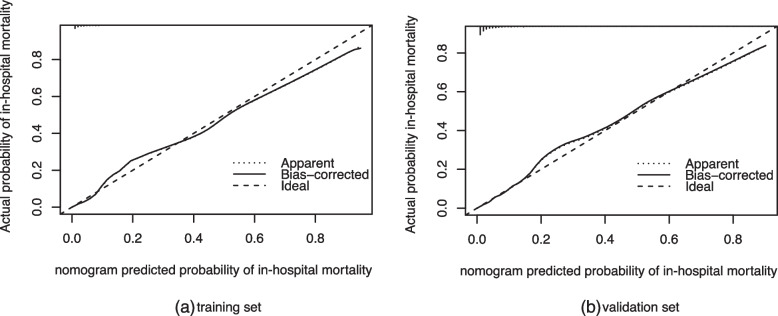
The calibration curve of the nomogram. **a** Training set, **b** validation set. The x-axis represents the in-hospital mortality risk predicted by the nomogram. The y-axis represents the actual in-hospital mortality rate. The diagonal dotted line represents the ideal prediction model. The solid line represents the prediction performance of the nomogram. The solid line is closer to the diagonal line, which shows that the nomogram has a higher degree of fit

**Fig. 5:**
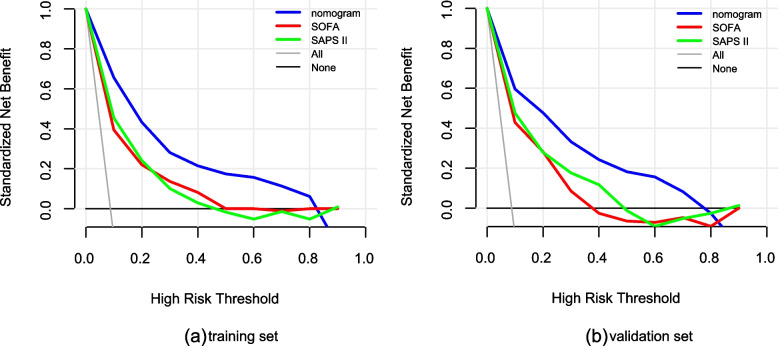
Decision curve analysis (DCA) of the training set and validation set. **a** Training set, **b** validation set. The y-axis measures the net benefit. The different colored lines represent the different predictive models of in-hospital death risk. The black solid line represents the assumption that all patients died in the hospital. The gray solid line represents the assumption that no patients died in the hospital. The decision curve shows that using this nomogram to predict in-hospital mortality adds more benefit than the SOFA and SAPS II scores (0.1 < *Pt* < 0.8)

In training set and validation set the calibration curve the nomogram demonstrated a high degree of fit (Fig. 4a, b). The clinical use of the nomogram in clinical application was compared with SOFA and SAPS II by DCA. In the training set and validation set, treatment directed by this nomogram gained more net benefit than SOFA and SAPS II when the threshold probability (*Pt*) > 0.1 and *Pt* <  0.8 (Fig. 5).

## Discussion

Nomogram is a graphic representation that consists of several lines. It is a pictorial representation of a complex mathematical formula. The numerical probability of individual clinical events can be generated by combining different prognostic variables and decisive variables [21]. Nomogram is widely used to predict prognoses in many diseases [22–24]. Nomogram has several advantages. First, it is user-friendly and could increase the accuracy of prediction. Second, it could help clinicians make better decisions. Recently, machine learning analysis was used to create a nomogram for predicting the prognosis of different patient cohorts [25, 26]. The clinical value of these predictive models lies in the identification of high-risk patients on one hand and the improvement in patient management through personalized medicine on the other. Most of them include multiple laboratory results, the results of coronary angiography, and echocardiogram parameters as predictive factors. The complexity of the factors limits their clinical application. This research has overcome these shortcomings. In our study, we attempted to establish a nomogram for predicting the in-hospital mortality of patients after PCI. It worth noting that the model in this study has a high AUROC and DCA in both the training and validation sets. In general, accurate prognostic assessment will help physicians intervene in a timely manner that balances risks and benefits.

Our study found that among patients after PCI, the risk of in-hospital mortality was higher in females than in males. This was consistent with previous studies [27–30]. One possible reason is that the symptoms of CHD in females are atypical, which delays the time to medical contact for female patients. A recent study showed that female patients are more likely to be complicated with coronary microvascular dysfunction, which may also be a reason for the higher risk of death [31].

Patients in ICU after PCI with lower systolic blood pressure, diastolic blood pressure and higher heart rate had a higher risk of in-hospital mortality. We suggested that the possible reason was that these patients were in shock or in a preshock state, and timely correction of shock or preshock might improve the prognosis of these patients. Patients in ICU who died in the hospital after PCI were more likely to be complicated with homeostasis disorders, including low bicarbonate, high potassium, and high anion gap. This suggests that metabolic acidosis was often present in patients with a high risk of death. Previous study has also shown an increased risk of death in critically ill patients with metabolic acidosis [32].

As indicated in some studies, higher WBCs and PT and lower hemoglobin and platelet counts have been investigated as risk factors for in-hospital mortality in patients after PCI. Indeed, as suggested by Liu et al. [33], we concluded that the elevation of WBCs on admission correlates with the in-hospital mortality of patients after PCI. Lower platelet counts, hemoglobin and higher PT may be related to bleeding after PCI. A previous study found that any amount of bleeding caused by PCI, including mild bleeding, was associated with worse outcomes [34]. However, due to the lack of data, we could not determine the real relationship between a higher PT, low platelet count, hemoglobin, and high risk of in-hospital death. More clinical research is needed in the future. CKMB can be used to estimate the extent of myocardial injury and is associated with the prognosis of CHD [35–37]. In our study, although there was no difference in CKMB levels between patients who died in the hospital and those who survived, it could still be observed that patients who died in the hospital had higher CKMB levels than those who survived. This difference might be because we included the value of CKMB at admission of patients after PCI. Not all patients undergo PCI because of AMI, some of them are stable angina, silent ischemia or after thrombolysis. They may in different stages of disease when they were admitted to the hospital. The CKMB at admission does not necessarily correspond to the peak of the CKMB or the severity of the disease.

Our results showed that the risk of in-hospital mortality was higher in patients with non-drug-eluting stents than in patients with drug-eluting stents. Previous studies have demonstrated that drug-eluting stents perform better than non-drug-eluting stents in the prevention of neointimal proliferation, restenosis, and associated clinical events [38–40].

We found that the risk of in-hospital death of patients who received IMV during hospital stay was higher than that of patients who received oxygen therapy or no oxygen treatment, which was consistent with our previous perception that patients with more severe illnesses are more likely to require mechanical ventilation [41]. However, we found that patients who received NIMV during hospital stay had a higher risk of in-hospital death than those who received IMV. In general, we think that patients with severe illness need to receive IMV rather than NIMV, which is somewhat contradictory to our previous perception. We speculate that the number of patients with IMV was small, would lead to bias.

There are some points that should be considered when using nomograms. Most of the laboratory results we used were within 24 hours after admission, so we need to obtain the relevant laboratory results as soon as possible after admission. Because most patients have multiple vital sign measurements after admission, we selected the average value of multiple vital sign measurements, which also needs to be considered.

There are several limitations of this study. First, the data for this study were extracted from MIMIC III, which is the database for a single center. Second, this study was a retrospective observational study, how the models identified in this study can be used for individualized evaluation and treatment of patients remains to be explored. Because of the unidentified confounding factors, the reliability of the results is lower than that of randomized, controlled clinical studies. Third, this study used data from the public database, and some of the data were not recorded. Even though we used multiple interpolation to fill in the missing data and appropriate calculation was based on this corrected data, there were still some differences from the original data. These differences might produce bias. Finally, the sample size of this study is still insufficient, and further studies may be needed.

## Conclusion

A nomogram was developed, and the AUROC of this nomogram was 0.907, which is excellent in predicting in-hospital mortality in critical ill patients after PCI. The nomogram revealed that it could augment net benefits in a wide range of threshold (0.1 < *Pt* <  0.8) in the prediction of the in-hospital mortality of patients after PCI.

## Supplementary Information

**Additional file 1.** Flowchart of data extraction.

**Additional file 2.** Baseline characteristics of training set.

**Additional file 3.** Baseline characteristics of validation set.

## Data Availability

The datasets used and analyzed during the current study are available from the corresponding author on reasonable request.

## Abbreviations

CHD: Coronary heart disease
PCI: Primary percutaneous coronary intervention
TIMI: Thrombolysis in myocardial infarction
AMI: Acute myocardial infarction
MIMIC III: Medical Information Mart for Intensive Care III
ICD-9: International Classification of Diseases-9
SOFA: Sequential Organ Failure Assessment
SAPS II: Scale for the Assessment of Positive Symptoms II
BMI: Body mass index
ICU: Intensive care unit
PT: Prothrombin time
WBC: White blood cell count
CKMB: MB isoenzyme of creatine kinase
TnI: Troponin I
TnT: Troponin T
LDL: l ow-density lipoprotein
TG: Triglyceride
Lac: Lactate
LASSO: Least absolute shrinkage and selection operator
OR: Odds ratio
ROC: Receiver operating characteristic
AUROC: Area under the receiver operating characteristic curve
NRI: Net reclassification improvement
IDI: Integrated discrimination improvement
DCA: Decision curve analysis
NIMV: Noninvasive mechanical ventilation
IMV: Invasive mechanical ventilation
