# Supplementary figures and images for: Development of a novel tool: a nomogram for predicting in-hospital mortality of patients in intensive care unit after percutaneous coronary intervention
Source: BMC Anesthesiol. 2023 Jan 6;23:5. doi: 10.1186/s12871-022-01923-y (PMC9817262; doi:10.1186/s12871-022-01923-y)

## Additional file 1 Flowchart of data extraction

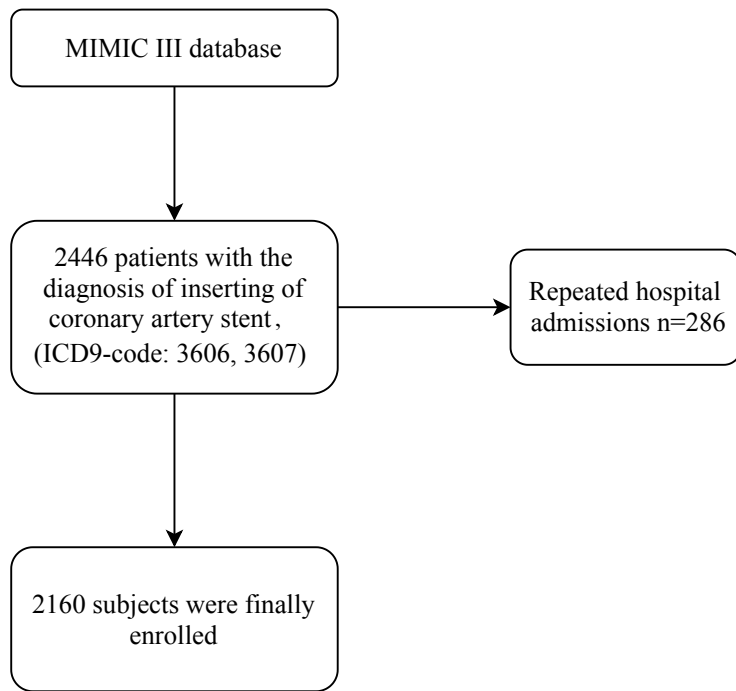

Supplement: Supplementary file 1 — Additional file 1. Flowchart of data extraction. [file 12871_2022_1923_MOESM1_ESM.pdf]
