# Supplementary material for: Development of a novel tool: a nomogram for predicting in-hospital mortality of patients in intensive care unit after percutaneous coronary intervention
Source: BMC Anesthesiol. 2023 Jan 6;23:5. doi: 10.1186/s12871-022-01923-y (PMC9817262; doi:10.1186/s12871-022-01923-y)
Supplement: Supplementary file 2 — Additional file 2. Baseline characteristics of training set. [file 12871_2022_1923_MOESM2_ESM.docx]

Additional file 2 Baseline characteristics of training set

| **Variable** | **Survive n=1181** | **Hospital mortality n=115** | **p-value** |
| --- | --- | --- | --- |
| Age(years) | 70(59-80) | 76(66-83) | p<0.001 |
| Gender(male/female) |  |  |  |
| Male | 774 | 65 | 0.053 |
| Female | 407 | 50 |  |
| Risk score |  |  |  |
| SOFA | 1(0-3) | 6(3-9) | p<0.001 |
| SAPS II | 30(23-37) | 47(37-57) | p<0.001 |
| Elixhauser comorbidity index | 0(0-5) | 5(0-10) | p<0.001 |
| Vital parameters |  |  |  |
| Systolic blood pressure (mmHg) | 113.19(104.28-124.67) | 102.62(92.39-114.31) | p<0.001 |
| Diastolic blood pressure (mmHg) | 59.67(53.04-67.01) | 54.05(47.56-60.06) | p<0.001 |
| Heart rate(min^-1^) | 76.12(67.95-85.65) | 86.22(74.96-102.17) | p<0.001 |
| Respiratory rate(min^-1^) | 17.88(16.14-20.04) | 19.05(16.66-22.40) | p<0.001 |
| Laboratory results |  |  |  |
| Hemoglobin (g/dL) | 11.9(10.4-13.3) | 10.8(9.65-11.95) | p<0.001 |
| Platelet (✖️10^9^/L) | 217.5(178.5-266.5) | 217(161-271) | 0.536 |
| Potassium (mmol/L) | 4.10(3.85-4.40) | 4.20(3.90-4.70) | 0.072 |
| Sodium (mmol/L) | 138(136-140) | 137.5(134-140) | 0.056 |
| PT (s) | 13.5(12.8-14.65) | 15(13.7-17.45) | p<0.001 |
| WBC (✖️10^9^/L) | 10.65(8.45-13.5) | 13.35(10.65-17.1) | p<0.001 |
| CKMB (mmol/L) | 68(15-198.5) | 88(22-204) | 0.166 |
| Anion gap (mmol/L) | 14(12-16) | 17(14-19) | p<0.001 |
| Bicarbonate (mmol/L) | 24(22-26) | 21(18-23) | p<0.001 |
| Chloride (mmol/L) | 104(101-106.5) | 104(100-108) | 0.587 |
| the type of coronary artery stent, n (%) |  |  | p<0.001 |
| Non-drug eluting stent | 588 (29.88%) | 85 |  |
| Drug-eluting stent | 593 (30.13%) | 30 |  |
| AMI, n (%) |  |  | 0.985 |
| Without the diagnose of AMI | 574 (29.17%) | 56 |  |
| With the diagnose of AMI | 607 (30.84%) | 59 |  |
| Ventilation treatment type, n (%) |  |  | p<0.001 |
| None | 121 (6.15%) | 2 (1.04%) |  |
| oxygen therapy | 1037 (52.69%) | 73 (38.02%) |  |
| NIMV | 17 (0.86%) | 38 (19.79%) |  |
| IMV | 6 (0.30%) | 2 1.04%) |  |
| Vasoactive drug, n (%) |  |  | p<0.001 |
| None | 783 (39.79%) | 23 (11.98%) |  |
| Vasopressin | 0 | 0 |  |
| Dobutamine | 8 (0.41%) | 0 |  |
| Epinephrine | 29 (1.47%) | 0 |  |
| Phenylephrine | 59 (3.00%) | 5 (2.60%) |  |
| Dopamine | 116 (5.89%) | 19 (9.90%) |  |
| Norepinephrine | 34 (1.73%) | 8 (4.17%) |  |
| Any two vasoactive drugs | 101 (5.13%) | 26 (13.54%) |  |
| Any three vasoactive drugs | 16 (0.81%) | 12(6.25%) |  |
| Four or more than four vasoactive drugs | 121 (1.78%) | 22 (11.46%) |  |

SOFA: sequential organ failure assessment

SAPS II: scale for assessment of positive symptoms II

PT: prothrombin time

WBC: white blood cell count

CKMB: MB isoenzyme of creatine kinase

AMI: acute myocadiac infraction

NIMV: noninvasive mechanical ventilation

IMV: invasive mechanical ventilation
