# Supplementary material for: Development of a novel tool: a nomogram for predicting in-hospital mortality of patients in intensive care unit after percutaneous coronary intervention
Source: BMC Anesthesiol. 2023 Jan 6;23:5. doi: 10.1186/s12871-022-01923-y (PMC9817262; doi:10.1186/s12871-022-01923-y)
Supplement: Supplementary file 3 — Additional file 3. Baseline characteristics of validation set. [file 12871_2022_1923_MOESM3_ESM.docx]

Additional file 3 Baseline characteristics of validation set

| **Variable** | **Survive n=787** | **Hospital mortality n=77** | **p-value** |
| --- | --- | --- | --- |
| Age(years) | 67(57-78) | 76(69-82) | p<0.001 |
| Gender(male/female) |  |  |  |
| Male | 498 | 37 | 0.009 |
| Female | 289 | 40 |  |
| Risk score |  |  |  |
| SOFA | 1(0-3) | 7(3.5-8.5) | p<0.001 |
| SAPS II | 29(22-36) | 49(37-62.5) | p<0.001 |
| Elixhauser comorbidity index | 0(0-5) | 5(3-10.5) | p<0.001 |
| Vital parameters |  |  |  |
| Systolic blood pressure (mmHg)) | 113.73(105.33-124.27) | 104.48(95.09-113.98) | p<0.001 |
| Diastolic blood pressure (mmHg) | 59.39(53.75-66.79) | 53.77(48.99-59.11) | p<0.001 |
| Heart rate(min^-1^) | 77(68.32-86.46) | 84.72(76.13-95.42) | p<0.001 |
| Respiratory rate(min^-1^) | 17.79(16.08-19.86) | 19.72(17.07-22.38) | p<0.001 |
| Laboratory results |  |  |  |
| Hemoglobin (g/dL) | 11.85(10.35-13.4) | 10.8(9.82-12.6) | p<0.001 |
| Platelet (✖️10^9^/L) | 221.5(183-274.5) | 199.5(153-265.75) | 0.016 |
| Potassium (mmol/L) | 4.10(3.85-4.40) | 4.30(1.0-4.8) | p<0.001 |
| Sodium (mmol/L) | 138.5(136.5-140) | 137(134-139.5) | 0.002 |
| PT (s) | 13.4(12.7-14.5) | 14.8(13.75-16.28) | p<0.001 |
| WBC (✖️10^9^/L) | 11(8.7-14.1) | 13.85(9.1-18.35) | p<0.001 |
| CKMB (mmol/L) | 70(21-215) | 61(12.5-250.5) | 0.831 |
| Anion gap (mmol/L) | 14(12-15.5) | 17(14.5-20) | p<0.001 |
| Bicarbonate (mmol/L) | 24(22.5-26) | 20(16.5-23) | p<0.001 |
| Chloride (mmol/L) | 104.5(101.5-107) | 104(100.25-107.5) | 0.567 |
| the type of coronary artery stent, n (%) |  |  | 0.001 |
| Non-drug eluting stent | 376 (47.78%) | 52 (67.53%) |  |
| Drug-eluting stent | 411 (52.22%) | 25 (32.48%) |  |
| AMI, n (%) |  |  | 0.478 |
| Without the diagnose of AMI | 366 (46.51%) | 39 (50.65%) |  |
| With the diagnose of AMI | 421 (53.49%) | 38 (49.35%) |  |
| Ventilation treatment type, n (%) |  |  | p<0.001 |
| None | 75 (9.53%) | 1 (1.30%) |  |
| oxygen therapy | 696 (88.44%) | 45 (58.44%) |  |
| NIMV | 11 (1.40%) | 31 (40.26%) |  |
| IMV | 5 (0.64%) | 0 |  |
| Vasoactive drug, n (%) |  |  | p<0.001 |
| None | 493 (62.64%) | 13 (16.88%) |  |
| Vasopressin | 1 (0.13%) | 0 |  |
| Dobutamine | 4 (0.51%) | 0 |  |
| Epinephrine | 16 (2.03%) | 0 |  |
| Phenylephrine | 47 (5.97%) | 5 (6.49%) |  |
| Dopamine | 74 (9.40%) | 8 (10.39%) |  |
| Norepinephrine | 27 (3.43%) | 8 (10.39%) |  |
| Any two vasoactive drugs | 87 (11.05%) | 25 (32.46%) |  |
| Any three vasoactive drugs | 16 (2.03%) | 8 (10.39%) |  |
| Four or more than four vasoactive drugs | 22 (2.80%) | 10 (12.99%) |  |

SOFA: sequential organ failure assessment

SAPS II: scale for assessment of positive symptoms II

PT: prothrombin time

WBC: white blood cell count

CKMB: MB isoenzyme of creatine kinase

AMI: acute myocadiac infraction

NIMV: noninvasive mechanical ventilation

IMV: invasive mechanical ventilation
